# Deep sequencing reveals an association between HIV-1 subtype C mutations in gp41 MPER epitopes and mother-to-child transmission

**DOI:** 10.1186/1742-4690-9-S2-P154

**Published:** 2012-09-13

**Authors:** L Yin, Y Cai, K Chang, BP Gardner, W Hou, K Nakamura, M Sinkala, C Kankasa, DM Thea, L Kuhn, GM Aldrovandi, MM Goodenow

**Affiliations:** 1 University of Florida, Gainesville, FL, USA; 2 Childrens Hospital of Los Angeles, Los Angeles, CA, USA; 3 Lusaka District Management Team, Lusaka, Zambia; 4 University Teaching Hospital, University of Zambia, Lusaka, Zambia; 5 Boston University School of Public Health, Boston, MA, USA; 6 Columbia University, New York, NY, USA; 7 Childrens Hospital of Los Angeles, Los Angeles, CA, USA

## Background

Enhanced HIV-1 mother-to-child transmission (MTCT) by high maternal anti-gp41 antibody titer led to the hypothesis that transmitting mothers would have greater diversity in membrane-proximal external region (MPER) and mutated amino acid residues associated with resistance to gp41 antibodies.

## Methods

Pyrosequences of HIV-1 subtype-C gp41 heptad repeat-region 2 (HR2), MPER, and membrane-spanning domain (MSD) were generated from 2,000 plasma viral copies/subject from four mothers who transmitted via breast feeding (TM) and four non-transmitting mothers (NTM) in a matched case control study. A bioinformatic pipeline with rigorous quality controls generated ~50,000 quality pyrosequences/subject and provided 25-fold coverage of input virus populations. Population genetic algorithms clustered pyrosequences at 3% genetic distance to study biodiversity using rarefaction/Chao1. Frequency distribution of cluster sizes defined population structure. Consensus sequences constructed from bioclusters for each subject were aligned to an HIV-1 subtype-C consensus sequence to determine number and frequency of nonsynonymous substitutions at each position and to identify mutations by HIV Molecular Immunology Database. Groups were compared using paired t-test.

## Results

Sequences in MPER were more polymorphic than in HR2 or in MSD. TM had more diverse MPERs than NTM (p = 0.02). The number of clusters calculated from rarefaction curves was 62(±35) for TM vs 35(±28) for NTM. The Chao1-estimated maximum number of variants within populations was 106(±51) for TM vs 59(±65) for NT. Low fit viruses (≤5 sequences/biocluster) contributed to differences in biodiversity between TM [71.1(±9.7)%] and NTM [63.5(±14.2)%]. Polymorphisms at residues within 4E10 (W672V, F673L, D674S, T676I, and W680G) and 2F5 (D664S) were confined exclusively to viruses from TM mothers. Viral variants with positively charged hydrophilic MPER occurred more frequently in TM than in NTM.

## Conclusion

HIV-1 subtype-C variants with high biodiversity correlated with polymorphisms in MPER and were associated with MTCT, which may reflect increased immune selection and have implications for vaccine design.

